# Paying Physicians to Prescribe Generic Drugs and Follow-On Biologics in the United States

**DOI:** 10.1371/journal.pmed.1001802

**Published:** 2015-03-17

**Authors:** Ameet Sarpatwari, Niteesh K. Choudhry, Jerry Avorn, Aaron S. Kesselheim

**Affiliations:** Program On Regulation, Therapeutics, And Law (PORTAL), Division of Pharmacoepidemiology and Pharmacoeconomics, Department of Medicine, Brigham and Women’s Hospital and Harvard Medical School, Boston, Massachusetts, United States of America

## Abstract

Aaron Kesselheim and colleagues examine potential strategies to promote greater prescribing of generic drugs and follow-on biologics

**Summary Points**

- Spending on brand-name prescription medications, particularly brand-name biologic drugs, continues to increase.
- Considerable savings and improvements in medication adherence are possible from greater use of more affordable generic drugs and imminently available follow-on biologics.
- Strategies to promote greater prescribing of generic drugs and follow-on biologics include traditional information-supplying programs such as formulary decision support and academic detailing and more novel financial incentives.
- While private insurers, qualified health plans, and employers may legally offer physicians financial incentives to prescribe generic drugs and follow-on biologics, marginally greater risks and a developing evidence base militates against physician payment for therapeutic or noninterchangeable follow-on biologic substitution, and—at this time—for bioequivalent substitution of narrow therapeutic index (NTI) drugs or interchangeable follow-on biologic substitution.
- Implementation of the various physician-centered strategies to promote greater prescribing of generic drug and follow-on biologics must be accompanied by comparative cost-utility studies assessing patient health outcomes.

## Introduction

Health care spending on prescription medications comprises 1.6% of gross domestic product (GDP) in the United States (US) and continues to rise [1]. Brand-name prescription medications—both small-molecule and biologic drugs—are the primary driver of this growth, increasing 15% in price in 2014 alone [2].

By contrast, low prices for generic small-molecule drugs (“generic drugs”), which now account for 86% of all prescriptions, have saved US consumers nearly $1.5 trillion in the past decade [3]. Much of this savings stems from substitution laws in every state that authorize or mandate pharmacists to fill most prescriptions for a brand-name drug with its generic counterpart and from tiered insurance formularies that impose higher cost-sharing obligations on patients for brand-name drugs.

Another potential source of health care savings in the near future may arise from the availability of follow-on biologic drugs. Generally derived from living cells, biologics account for less than 1% of all prescriptions but currently make up about 28% of prescription drug spending in the US [4]. In 2009, Congress authorized the Food and Drug Administration (FDA) to abbreviate the approval process for biosimilar versions of biologics, enabling manufacturers of these “follow-on” products to forgo conducting the same costly clinical trials required for the innovator products. Nineteen follow-on biologics are available in the European Union (EU) and cost on average 20%–25% less than their innovator counterparts (Table 1) [5,6]. In 2014, the FDA received its first two follow-on biologic applications (for rituximab and filgrastim) [7]. While both applications remain under review, an FDA scientific advisory committee has recently recommended approving Sandoz’s follow-on filgrastim [8].

**Table 1:** Approved follow-on biologics in the EU.

Active Ingredient	Brand Name	Manufacturer	Indication	Approval Year
epoetin alfa	Abseamed	Medice Arzneimittel Pütter	anemia associated with chemotherapy or chronic renal failure	2007
	Binocrit	Sandoz		2007
	Epoetin Alfa Hexal	Hexal		2007
	Retacrit	Hospira		2007
	Silapo	Stada Arzneimittel		2007
filgrastim	Accofil	Accord Healthcare	neutropenia associated with bone marrow transplantation. chemotherapy, or HIV infection; congenital or idiopathic neutropenia	2014
	Biograstim	AbZ-Pharma		2008
	Filgrastim Hexal	Hexal		2009
	Grastofil	Apotex		2013
	Nivestim	Hospira		2010
	Ratiograstim	Ratiopharm		2008
	Tevagrastim	Teva		2008
	Zarzio	Sandoz		2009
folitropin alfa	Bemfola	Finox Biotech	anovulation; hypogonadotropic hypogonadism	2014
	Ovaleap	Teva		2013
infliximab	Inflectra	Hospira	rheumatoid arthritis	2013
	Remsima	Celltrion		2013
insulin	Abasaglar	Eli Lilly	diabetes	2014
somatropin	Omnitrope	Sandoz	adolescent growth disturbances	2006

To achieve even greater use of generic drugs and capitalize on possible savings from the imminent availability of follow-on biologics, many US policymakers and payers support supplementing state substitution laws and tiered formularies with more widespread use of physician-focused initiatives. These programs have traditionally supplied enhanced information, as with formulary decision support and academic detailing. More recently, however, insurers and employers have sought to provide financial incentives to physicians. In this article, we review clinical, legal, and ethical questions surrounding the use of traditional physician-centered programs and more novel pay-for-performance strategies to promote generic drugs and evaluate their applicability to follow-on biologics.

## Types of Substitution

### Bioequivalent Substitution

While physicians can directly initiate prescriptions for generic drugs, most generic drug use arises from two varieties of substitution: bioequivalent and therapeutic. In bioequivalent substitution, a brand-name drug is replaced with an interchangeable generic drug possessing the same amount of chemically equivalent active ingredient and comparable activity at a target site. To meet this standard, the FDA requires a demonstration that the 90% confidence intervals for brand-to-generic ratios of the active ingredient’s maximum serum concentration (C_max_) and area under the serum concentration over time curve (AUC) fall within 0.80 to 1.25, the so-called “−20/+25 rule” [9].

Some drug safety experts have argued that the −20/+25 rule is inadequate for narrow therapeutic index (NTI) drugs—drugs for which a small change in dose “produces clinically significant and undesirable … increased adverse effects, decreased therapeutic efficacy, or excessive therapeutic effects” [10]. Case reports and observational studies have reported that bioequivalent NTI drugs may not have the same clinical effects as their brand-name counterparts [11–13]. These results, however, have been contradicted by more well-controlled studies that found no evidence that bioequivalent substitution was associated with exacerbations of disease [14–16], prompting the American Medical Association Council on Science and Public Health to report that evidence of clinical differences between brand-name NTI drugs and their bioequivalent generics “either does not exist or is extremely weak” [17]. Indeed, the FDA’s bioequivalence requirements are often surpassed by generic drug manufacturers. A comprehensive review of generic drugs approved by the FDA between 1996 and 2007 found that C_max_ and AUC values differed on average by 4.4% and 3.6%, respectively, compared to their brand-name counterparts; 98% of these values were less than 10% [18]. Although rare cases of substandard products being withdrawn from the market exist [19,20], such products are not limited to generic drugs [21].

Pharmacist substitution of brand-name drugs with their FDA-approved bioequivalent generic counterparts is common, generally occurring automatically through state laws applicable to private and public payers. These laws, however, can be bypassed by the active intervention of a patient or physician. Twenty-one states, for example, require patient consent prior to pharmacist-driven substitution of a bioequivalent product. In addition, physicians can write prescriptions for brand-name drugs that are specifically marked as “dispense as written.” A 2011 study found that physicians and patients directed pharmacists to dispense as written nearly 5% of prescriptions [22], actions contributing to an additional US$1,200 million in excess drug costs annually in the US [23].

### Therapeutic Substitution

The second type of generic drug substitution is therapeutic substitution, which involves products that are not bioequivalent. Therapeutic interchange is possible because many classes of drugs contain multiple chemically-related but distinct drugs that share a common mechanism of action. For example, there are currently eight types of angiotensin-II-receptor blockers (ARBs) on the US market. Therapeutic substitution from a brand-name ARB like olmesartan (Benicar) to a generically-available ARB like telmisartan (Micardis) can reduce spending and may represent good value in patients for whom brand-name drugs do not offer significant benefits over and above generics in the same class. As with bioequivalent substitution, large savings remain possible from increased therapeutic substitution. An evaluation by the nonprofit news organization ProPublica found that altering the prescribing of just 913 physicians to reflect common therapeutic substitution practices would have saved taxpayers US$300 million in 2011 [24].

### Follow-On Biologic Substitution

Abbreviated approval of follow-on biologics in the US was initially authorized by the Biologics Price Competition and Innovations Act of 2009 (BPCIA). Follow-on biologics are required to have the same mechanism of action, route of administration, and dosage form as the innovator and not be meaningfully different with regard to safety, purity, or potency [25]. The FDA has thus far issued four draft guidelines on meeting these requirements [26]. When follow-on biologics are approved by the FDA, a select few will be deemed interchangeable, while most will simply be approved as members of the same drug class to treat the same conditions. In this respect, interchangeable and noninterchangeable follow-on biologic substitutions are roughly analogous to bioequivalent and therapeutic substitutions, respectively.

Saving prohibiting the use of an unapproved innovator biologic as a reference product and enumerating a pathway for formal declaration of interchangeability [27], the scientific standards put forth by the FDA about follow-on biologics largely mirror those adopted by the European Medicines Agency (EMA), which has a longer track record with such drugs [28]. Despite the European experience, however, concerns about follow-on biologics remain, including difficulties with establishing the similarity of complex molecules made by different manufacturers and the possibility that even small variations in follow-on biologic products may induce immune responses [29,30]. These complexities have hindered follow-on biologic development, spurring greater investment in brand-name biologics relative to small-molecule drugs, and have enabled brand-name manufacturers to successfully lobby three states to increase barriers for pharmacists to automatically substitute innovator biologics with follow-on products [31–34]. Some professional societies, patient groups, and innovator manufacturers, meanwhile, have urged the FDA to adopt naming conventions that clearly distinguish follow-on biologics from innovator products [35,36].

## Physician-Centered Strategies to Promote Generic Drug Prescribing

### Traditional Approaches

Information-supplying programs have long been employed by insurers and employers to encourage physician prescribing of generic drugs. Formulary decision support provides physicians with disease management guidelines and patients’ insurance formularies at the point of prescribing to facilitate a choice of medication that is safe, effective, and affordable. In one study involving 1.5 million patients and 35,000 physicians, investigators found a 3.3% absolute increase in generic drug prescribing by physicians given a color-coded breakdown (green, blue, and red) of preferred formulary (i.e., generic), nonpreferred formulary, and nonformulary medications, translating to an estimated savings of US$845,000 in prescription drug costs per 100,000 people [37].

Academic detailing capitalizes on dissemination techniques used by brand-name drug manufacturers, supplying physicians with individually tailored information on optimal prescription drug use through in-person, interactive sessions with trained educators at physicians’ offices [38]. As opposed to brand-name drug sales representatives, however, academic detailers seek to present physicians with neutral, evidence-based educational materials on cost-effective practices developed by university-based experts. Academic detailing has been shown to have a positive influence on physician prescribing practices. In a meta-analysis of 28 studies, the Cochrane Collaboration reported a 5.6% median risk difference in compliance with desired practices between physician recipients of academic detailing and controls, leading to the conclusion that such programs “have effects on prescribing that are relatively consistent and small, but potentially important” [39].

### Financial Incentives

More recently, direct financial incentives have been used to promote generic drug prescribing. In 2007, the Blue Care Network in Michigan ran a 90-day program that offered physicians US$100 for every patient switched from a brand-name to a generic cholesterol-lowering medication [40]. In New York, Excellus BlueCross BlueShield provided a higher office visit payment rate for physician groups that increased their generic drug fill rates by 5% or more, while Independent Health paid physicians who prescribed 70% or more generic drugs monthly bonuses of $US0.50 per patient seen [41]. Although the programs were not subject to independent scientific evaluation, the Blue Care Network stated that its 90-day promotion cost US$2 million but saved the company US$5 million in drug costs and patients US$1 million in copays in just five months. Excellus BlueCross BlueShield estimated that its pilot reduced patients’ out-of-pocket costs by 10%–12% [40]. A 2010 national survey of pay-for-performance programs found that it is commonplace for physicians to earn bonus payments from payers for generic drug prescribing in the US [42]. In California, for example, seven insurers use a common physician performance measure that includes generic drug prescribing for several common conditions like hypertension and depression [43].

## Discussion

### Legal Considerations

Some concerned groups have argued that payments for generic drug prescribing expose physicians to liability under the federal anti-kickback statute [44], which prohibits offering or soliciting payment to induce the purchase of items paid for by a federal health care program [45]. As Medicare Part D and Medicaid are programs that provide federal payment for prescription medications for the elderly and indigent, respectively, the anti-kickback statute clearly precludes Medicare Part D insurance providers and Medicaid managed care organizations from offering physicians financial incentives to prescribe generic drugs or follow-on biologics. Its reach beyond these programs, though, is limited in two respects. First, the anti-kickback statute exempts “any amount paid by an employer to an employee” so hospitals and managed care organizations that employ physicians can safely incorporate generic drug and follow-on biologic prescribing as a measure in their pay-for-performance schemes. Second, the anti-kickback statute does not prohibit physicians from accepting prescribing bonuses from private health plans. In October 2013, the Department of Health and Human Services stated that it did not consider health plans offered through the Patient Protection and Affordable Care Act’s new insurance exchanges to be federal health care programs [46]. Thus, there are still many situations in which physicians can accept financial incentives for prescribing generic drugs or follow-on biologics.

### Ethical Considerations

The fact that physicians may be offered bonuses by private insurers, qualified health plans, and employers for prescribing generic drugs and follow-on biologics does not imply that they should accept them. After all, inducements to encourage brand-name drug prescribing have been the subject of substantial controversy, having been shown to result in treatment-naïve patients commencing expensive and potentially dangerous medication therapy that was not indicated and patients switching to the promoted brand-name drug instead of following evidence-based guidelines [47].

Financial incentives for prescribing generic drugs, however, do not raise the same set of concerns. Many brand-name drugs are now approved based on limited premarket testing, which can expose patients to unknown risks [48]. By contrast, since generic drugs have been in wide use longer than brand-name drugs, their benefit-to-risk ratios tend to be better characterized [49]. Physicians prescribing generic drugs, and patients taking them, are therefore more likely to know how to optimize their safe use. The lower prices of generic drugs, moreover, promote adherence to essential medications when shouldered by patients [50]. A study of six classes of chronic medications between 2001 and 2003 found 12.6% greater adherence among US patients who commenced treatment with a generic rather than a brand-name drug [51]. These differences support paying physicians to promote generic drug prescribing.

### Authors’ Recommendations

The distortive impact that financial incentives could have in clouding clinical decision making with the prospect of profit, though, suggests that policymakers should be circumspect about the situations in which they are used. Financial incentives are generally not appropriate for therapeutic or noninterchangeable follow-on biologic substitution (Table 2). Generic drugs used in therapeutic substitution have different active ingredients than the innovator product, and noninterchangeable follow-on biologics need not demonstrate clinical equivalence to the innovator product. Therapeutic and noninterchangeable follow-on biologic substitutions therefore expose patients to marginally greater risk than bioequivalent and interchangeable follow-on biologic substitutions. Financial incentives should also not be offered to physicians for initiating treatment-naïve patients on generic drugs or follow-on biologics, as payments could prompt unnecessary treatment or preclude clinically preferable treatment with a nonbioequivalent brand-name drug.

**Table 2:** Authors’ evaluation of physician-centered strategies to promote generic drug and follow-on biologic prescribing ^*^

Type of program	Traditional strategies	Direct financial incentives
**Example**	Formulary support and academic detailing	US$100 for every prescription switched
**Appropriateness as applied to**		
**Small-molecule drugs**		
**Bioequivalent substitution**	Recommended	Recommended
**Bioequivalent substitution of narrow therapeutic index drugs**	Recommended	Not recommended ^*^
**Therapeutic substitution**	Recommended	Not recommended
**Biologics**		
**Interchangeable follow-on biologic substitution**	Recommended	Not recommended^*^
**Noninterchangeable follow-on biologic substitution**	Recommended	Not recommended

^*^ Pending further evidence.

Instead, physician payments should be limited to bioequivalent substitution of non-NTI drugs. Further comparative effectiveness research studies are currently being organized to evaluate generic NTI drugs [52], and the evidence base for follow-on biologics continues to evolve. Given this knowledge, it would be prudent not to extend financial incentives to bioequivalent substitution of NTI drugs or to interchangeable follow-on biologic substitution until there is more widely accepted assurance of safety and effectiveness in the US market.

Notwithstanding limitations imposed by NTI drugs and the anti-kickback statute, substantial savings from financial incentives for generic and follow-on biologic prescribing remain possible. In 2013, 52% (164 million) of the US population had private health insurance [53], and over 6 million people have signed up for coverage through the new insurance exchanges [54]. Combined, these 170 million people represent a majority of the nationwide cohort from which US$1,200 million in annual savings from increased bioequivalent substitution can be secured. As physicians who care for these patients also typically treat Medicare Part D and Medicaid patients, private insurer- or employer-based programs might have spillover effects for public payers. However, considerable research is still needed to determine outcomes from these programs. In particular, comparative cost-utility data on various payment models and on pay-for-performance generally relative to more traditional information-supplying programs are needed, factoring patient health outcomes in addition to cost savings. Such information could play a critical role in incentivizing private insurers and employers to design and implement physician-centered interventions to promote generic drug and follow-on biologic prescribing on a wider scale.

To encourage treatment initiation on generic drugs or follow-on biologics, therapeutic or noninterchangeable follow-on biologic substitution, or bioequivalent NTI or interchangeable follow-on biologic substitution, policymakers can instead rely on traditional strategies, which remain underused. Formulary support and academic detailing can improve decision making and help physicians overcome the habit of thinking about and referring to drugs by their brand-names as well as the false perception that generic drugs are less effective than their brand-name counterparts [55]. In a 2011 survey, however, just 34% of office-based physicians reported even having a basic electronic health record system through which effective formulary support can be implemented [56], and only a handful of state governments sponsor academic detailing initiatives [57]. Incentives should be considered to encourage insurers to develop formulary applications that can work with common electronic health record systems, which are already being promoted by the federal government to implement meaningful use standards. Even the incorporation of so-called nudges—subtle interventions that “change people’s behavior in a predictable way without forbidding any options or significantly changing their economic incentives” [58]—within traditional physician-centered strategies poses less risk of undue influence than bonuses. Implementation of an e-prescribing system that returns physician-initiated searches for a medication with lists programmed to place generic drug first in larger and bolder font [59], for example, can correct for inherent cognitive biases like choice overload—the likelihood that physicians will maintain the status quo when presented with many, rather than few, alternatives [60]—in addition to countering habits and false perceptions without changing the cost–benefit calculus of drug selections. More research is needed to develop and test a wider spectrum of nudges like default generation of e-prescriptions for interchangeable generic drugs or follow-on biologics when available and publication of physicians’ generic drug and follow-on biologic prescribing rates.

## Conclusion

Generic drugs and follow-on biologics can improve patient health outcomes and reduce health care spending. Physician-centered tools to promote their prescribing include information-supplying programs and financial payments. Both interventions can be safely, legally, and ethically employed by private insurers and employers to encourage bioequivalent substitution of non-NTI generic drugs. To prevent unnecessary or suboptimal treatment, however, information-supplying programs like formulary decision support or academic detailing alone should be used to enhance therapeutic or noninterchangeable follow-on biologic substitution, treatment initiation with generic drugs or follow-on biologics, and—until further safety and effectiveness data are available—bioequivalent NTI or interchangeable follow-on biologic substitution.

## Abbreviations

ARBs: angiotensin-II-receptor blockers
AUC: area under the serum concentration over time curve
BPCIA: Biologics Price Competition and Innovations Act
EMA: European Medicines Agency; EU, European Union
FDA: Food and Drug Administration
GDP: gross domestic product
NTI: narrow therapeutic index
US: United States
